# Quality and Safety Risk Control in the Food Supply Chain: An Information Disclosure Approach to Supply–Demand Alignment

**DOI:** 10.3390/foods15050876

**Published:** 2026-03-04

**Authors:** Menghui Qiu, Yun Luo, Taiping Li

**Affiliations:** 1 Rural Development Institute, Yan’an University, Yan’an 716000, China; qingshanyuwl@163.com; 2 College of Economics and Management, Nanjing Agricultural University, Nanjing 210095, China

**Keywords:** food safety social co-governance, food inspection information, supply and demand matching, supply chain, market-driven mechanism

## Abstract

The government’s scientific disclosure of food safety inspection information can guide consumers toward rational substitution choices, thereby improving food safety while transforming individual decision-making into collective action, thereby achieving social co-governance. This process activates the “voting with their feet” market mechanism, which exerts pressure on supply chain enterprises to improve quality control. However, the current mismatch between disclosed information and consumer demand significantly weakens this effect. Drawing on evolutionary game theory, this study constructs an evolutionary game model involving producers, sellers, and consumers to explore how information alignment shapes stakeholder behavior. The findings indicate that improving information alignment effectively nudges consumers toward informed substitution choices, reinforcing the market-driven pressure on supply chain enterprises to strengthen quality control; reducing quality control costs is a more effective short-term incentive for sellers than increasing market returns; and when information alignment is low, prioritizing inspections of sellers more efficiently enhances co-governance performance, whereas under high alignment, stronger regulation of producers becomes more effective. Aligning the content, channels, and presentation of government-disclosed inspection information with consumer needs is critical to empowering effective social co-governance. These findings provide theoretical foundations and policy insights to optimize information disclosure strategies and regulatory resource allocation.

## 1. Introduction

Lack of food safety poses a significant threat to public health. Unsafe food is estimated to cause 600 million cases of food-borne illness and 420,000 deaths annually worldwide [1], making it an urgent challenge for China and other countries. Although China’s food safety conditions have improved in recent years, incidents continue to be reported. Media-exposed cases such as the “earth-pit” pickled cabbage scandal (https://www.samr.gov.cn/xw/mtjj/art/2024/art_fb70e0b095944502b19a7c7146dae009.html (accessed on 16 January 2026)) and the “rat head in packaged duck neck products” (https://app.people.cn/h5/detail/normal/5328450048885760 (accessed on 16 January 2026)) incident have heightened public concern. Addressing food safety risks, therefore, remains a pressing policy challenge. In response, the Chinese government has adopted a range of regulatory instruments, including sampling inspections and traceability systems [2,3,4]. Nevertheless, these efforts continue to face a persistent “regulatory dilemma” [5]. Against this backdrop, collaborative governance involving the public, enterprises, and the media has emerged as an internationally recognized model for food safety management [6,7].

From an economic perspective, food safety problems stem from information asymmetry among supply chain actors [8]. In China, most incidents occur during the production and processing stages [4]. Strengthening quality management across the supply chain is therefore essential, requiring both effective government oversight and coordinated market incentives [9,10]. Within a social co-governance framework, consumers can drive safer production by rewarding trustworthy products and reshaping firms’ profit structures [11,12]. Sellers—distributors and retailers—serve as critical intermediaries and, if they rigorously screen non-compliant products, can effectively block quality risks along the supply chain [7,13] (according to the *Supervision and Management Measures for Food Safety in Distribution Links*, qualified food enterprises, including wholesale markets and supermarkets, are required to conduct self-inspections). However, public participation in social co-governance is mainly through consumer complaints and whistleblowing, which are costly and often suffer from a “high willingness, low action” gap [14]. Persistent information asymmetry [15,16] undermines consumers’ ability to enforce quality, reducing incentives for sellers and producers to invest in additional quality control and resulting in systemic governance failures.

The disclosure of food safety inspection information has become a novel regulatory tool for addressing information asymmetry and enhancing food safety governance. Based on nudge theory, governments can promote public welfare through non-coercive interventions that preserve individual autonomy by designing effective “choice architectures” [17]. In China’s diverse food market, where products are highly substitutable, consumers with varying income levels and risk preferences can make rational substitution choices. According to the State Administration for Market Regulation, annual inspection reports rose from 1.50 million in 2016 to 6.99 million in 2023. These data reveal differences in risk levels across brands, categories, origins, sales channels, and sellers [18]. Through random inspections of 34 food categories across production, distribution, and catering, the government identifies non-compliant enterprises and discloses results. Public disclosure of such risk information enables consumers to select products with higher qualified rates, nudging them toward more rational purchasing behavior [17,19]. In this way, dispersed consumer choices generate collective market signals, prompting sellers and producers to strengthen quality control and reinforcing the market’s role in food safety governance.

Nevertheless, the effectiveness of information disclosure is often limited for both consumers and food enterprises [20,21,22]. Although prior studies find that disclosure can reduce demand for unsafe products [23,24,25,26,27] and motivate firms to improve quality control [2,28,29,30], these effects are often short-lived. Market responses to negative food safety information tend to diminish over time [26,31,32]. This limited impact stems in part from the conditional nature of nudging, which depends on factors such as consumer perceptions, preferences, information structures, and cognitive capacity [33,34]. In China, misalignments between the content, channels, and presentation of disclosed information and consumer needs further reduce its utility [35,36,37], leading to low engagement [38,39]. Without accessible and actionable information, consumers struggle to make informed substitution choices, weakening the nudging effect and hindering coordinated quality governance along the supply chain.

In recent years, an expanding body of research has explored how information disclosure can strengthen social co-governance in food safety. However, most studies rest on the implicit assumption that disclosure alone ensures effectiveness, which limits their practical relevance. In practice, deficiencies remain in the design and implementation of disclosure systems. Critical questions arise: What weaknesses exist in governmental information disclosure? How can disclosure mechanisms be made more effective in guiding consumers toward rational substitution decisions, motivating sellers to voluntarily test and select high-quality products, and thereby enabling genuine social co-governance? To address these questions, this study develops a three-stage evolutionary game model encompassing producers, sellers, and consumers within a social co-governance framework. Centered on the effectiveness of governmental information disclosure, the model systematically characterizes the behavioral evolution of these actors under different disclosure conditions. It identifies key factors shaping quality control incentives and co-governance participation and offers theoretical insights for optimizing food safety inspection information disclosure.

The marginal contributions of this study are threefold. First, unlike prior studies emphasizing public participation through complaint or reporting mechanisms, this study examines how effective disclosure of food inspection information can guide consumers toward informed substitution strategies. By improving their own food safety outcomes at minimal participation costs, consumers are naturally integrated into social co-governance, thereby enriching mechanisms of public involvement. Second, this study emphasizes the pivotal intermediary role of sellers in the supply chain and investigates the spillover effects of information disclosure across upstream and downstream enterprises, thereby expanding the scope of research on its impact within the food supply chain. Third, numerical simulations reveal that reducing quality control costs provides stronger short-term incentives for sellers than increasing product premiums, offering theoretical insights for evidence-based policy design.

## 2. Research Hypothesis

### 2.1. Definition of Concepts

(1) Disclosure of food sampling information. This study distinguishes between two types of food safety sampling: government-led inspections and seller-initiated testing. Government sampling, conducted by market regulatory authorities, aims to identify non-compliant products and enterprises in the production and distribution stages, with disclosed results guiding consumers toward safer choices. In contrast, seller-led sampling—such as self-testing by supermarkets or wholesalers—focuses on internal quality management to reduce operational risks. Correspondingly, information disclosure takes two forms: (1) public disclosure of government sampling results, such as bulletins from the State Administration for Market Regulation (SAMR) covering wide regions and large sample sizes, and (2) seller disclosure of self-testing results, typically posted in retail or wholesale venues for internal control purposes.
(2) Mismatch between information supply and demand. This study defines the mismatch between information supply and demand as the gap between food sampling results disclosed by governments or sellers and consumers’ actual food safety information needs. This gap may appear in content (e.g., reports focus on failed products, while consumers seek safe alternatives), channels (e.g., official websites vs. consumer reliance on media), and presentation (e.g., complex tables vs. clear, user-friendly formats). As a result, the disclosed information often fails to meet consumer needs, limiting its usefulness and impact.
(3) Scientific consumption substitution. This concept refers to consumers’ proactive adjustment of purchasing choices based on government-disclosed food safety inspection results. By systematically comparing food safety risk levels across brands, product categories, places of origin, sales channels, sellers, and other relevant attributes, consumers make more informed assessments of food safety risks. They then substitute foods with lower compliance rates and higher risks with those exhibiting higher compliance and lower risks, leading to more rational and safer consumption decisions.

### 2.2. Description of the Problem

**Figure 1:**
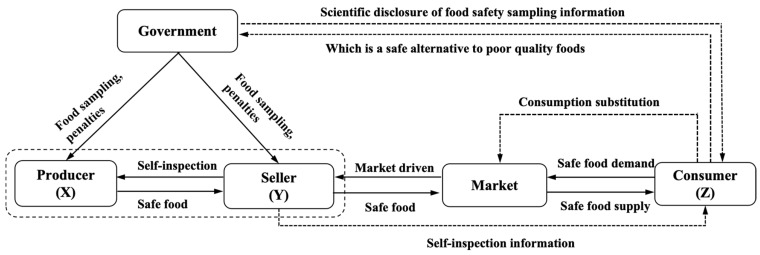
A quality control mechanism for the food supply chain based on the matching of supply and demand for inspection information disclosure under the social co-governance framework.

The food supply chain is highly complex. To simplify the analysis, this paper abstracts the food supply chain system into a tripartite evolutionary game model, consisting of upstream producers (X), sellers (Y), and consumers (Z). Using this model, the study demonstrates how government disclosure of food sampling information influences the strategic choices of these entities, ultimately advancing social co-governance in food safety. To visually demonstrate the interactions among the key actors in the food supply chain, the paper provides an operational schematic based on government food sampling and market feedback mechanisms (see Figure 1). In this model, producers (X) and sellers (Y) aim to maximize profits, while consumers (Z) seek utility maximization, with all players assumed to be boundedly rational. Each player is further assumed to have two strategic options. The assumptions are outlined below, with relevant parameters listed in Figure 1.

### 2.3. Modelling

To further analyze the formation mechanism of social co-governance in food safety, the following hypotheses are proposed. Definitions of all model parameters are summarized in Table 1.

**Assumption** **1.** *For producer X, let the profit from producing safe food be* $M_{H}$ *and the abnormal profit from producing substandard food be* $M_{L}$*. Safe production generally requires stricter raw material selection, process monitoring, and compliance measures, which incur additional quality control costs. In a fixed-price market, producing substandard food and thereby avoiding these quality obligations reduces costs and increases net profit, so that* $M_{L}>M_{H}$ *[7]. Without a profit differential, producers have little incentive to engage in substandard production. Suppose the government’s regulatory agency conducts sampling inspections with a probability of* $g_{m}$*, and if any violations are detected, X incurs a penalty of* $P_{GM}$. *Given that the government discloses non-compliance results to consumers, X also faces market penalties of* $P_{CM}$ *through consumer “voting with their feet”. To ensure food safety, seller Y conducts inspections on the products supplied by X, with the effective inspection rate,* b(w,e) *(drawing on the concept of “effective sampling rate” proposed by Wan et al. (2018) [40], this rate represents the probability that Y detects non-compliance when inspecting food supplied by X, provided X has delivered substandard products), determined by Y’s inspection willingness,* w*, and equipment investment,* e*, where* 0≤b(w,e)≤1*,* 0≤w≤1*, and* 0≤e≤1 *When X chooses to produce substandard food, and Y conducts inspections, X incurs an additional fine of* $b\times P_{SM}$*. Based on the above analysis, if X chooses to produce substandard food, Y enforces quality control, and consumers make informed substitutions, X’s final profit will be* $M_{L}-g_{m}\times \left(P_{GM}+P_{CM}\right)-b\times P_{SM}$.

**Assumption** **2.** *For seller Y, let the revenue from implementing quality control be* $S_{H}$ *and the revenue without quality control be* $S_{L}$*. Since quality control incurs additional costs, we have* $S_{L}$ *>* $S_{H}$ *[7]. Let* $C_{M}$ *denote the cost for Y to implement quality control on sold products, including sampling and testing. By conducting inspections and publicly disclosing the results, Y can earn not only the standard profit but also additional market returns driven by consumer preferences for food safety information, such as price premiums or increased sales [41,42]. These gains are collectively termed a “market reward,” denoted by* m*, representing benefits from price premiums, sales growth, or increased market share. Thus, Y’s total profit in this scenario becomes* $S_{H}-C_{M}+m$*. If seller Y implements quality control while the supplier (X) provides risky products, Y may impose a penalty,* $b\times P_{SM}$*, on X but incurs testing costs,* $C_{M}$*, yielding a net gain of* $b\times P_{SM}-C_{M}$*. In such cases, Y can switch to alternative, compliant suppliers. Assuming the substituted products meet safety standards, Y retains a profit of* $S_{H}-C_{M}+m$*. Consequently, Y’s potential aggregate profit under this case amounts to* $S_{H}-{2C}_{M}+b\times P_{SM}+m$*. If Y opts not to implement quality control, there is a probability,* $g_{s}$*, that government regulators will detect the substandard food, leading to a penalty of* $P_{GS}$*. Furthermore, when the government discloses information on inspection failures, consumers may shift their purchases, resulting in market losses for Y, denoted as* $P_{CS}$*. Therefore, Y’s profit from selling substandard food without conducting inspections is* $S_{L}-g_{S}\times \left(P_{GS}+P_{CS}\right)$.

**Assumption** **3.** *Consumer Z seeks to maximize consumption utility. As utility is difficult to measure directly, this study adopts the approach of Wu et al. (2024) [4], using consumer surplus as a proxy for Z’s benefit. (According to consumer behavior theory, utility is maximized when the marginal utility a consumer gains from purchasing a unit of a good equals the marginal monetary utility they pay. Given that marginal utility decreases while marginal monetary utility is often assumed to be constant, consumers obtain a “consumer surplus.” This surplus represents the difference between the maximum total price the consumer is willing to pay for the good and the actual price paid.) When Z engages in scientific consumption substitution—indicating a higher demand for food safety and willingness to pay—their consumer surplus is denoted as* S1*. This substitution requires Z to access and compare safety information across food alternatives, incurring costs related to information acquisition, processing, and opportunity loss. Let* a *(where* 0≤a≤1*) represent the degree of alignment between government-disclosed information and Z’s demand. When the information disclosed by the government is completely misaligned with consumer demand (*a=0*), and sellers do not conduct food safety inspections, the cost incurred by consumers to obtain food quality information that meets their own needs is denoted as* C1*. If sellers conduct food safety inspections and publicly disclose the inspection results, the transparency of seller-level inspection information reduces the difficulty of information collection, processing, and evaluation (e.g., in terms of time required) for consumers, thereby lowering the cost of acquiring demand-relevant information, denoted as* C2 *(*C1>C2*). Accordingly, when sellers do not conduct food safety inspections, the cost for consumers to obtain substitute food quality information is* (1−a)×C1*; when sellers conduct food safety inspections and disclose the results, the corresponding cost is* (1−a)×C2 *(assuming that Y and the government’s information alignment with Z’s demand are equivalent).*

If producers (X) supply unsafe food, Z will switch to safer alternatives, incurring a switching cost, C3, due to habitual preferences. Thus, when Y implements quality control, Z’s net utility is S1−(1−a)×C2−C3, and when Y neglects quality control, Z’s net gain from scientific substitution is S1−(1−a)×C1−C3. If Z does not engage in scientific consumption substitution, their willingness to pay for safer food is presumed lower, reflected by a reduced consumer surplus, S2. Despite this lower valuation, Z remains risk-averse; thus, the expected surplus when consuming risky food remains S2. Since substitution yields safer alternatives, utility theory dictates S1>S2. The tripartite game payoff matrix among producers, sellers (Y), and consumers (Z) is detailed in Table 2.

## 3. Model Analysis

### 3.1. Analysis of the Evolutionary Strategies of Producers

Here, $E_{11}$, $E_{12}$, and $E_{1}$ represent expected profits for the supply of safe food and substandard food and the average expected profit of X, respectively. The replicated dynamic equation for the selection of a strategy for X behaviors is F(x), and Equations (1)–(3) may result from Table 2.

(1) $$E_{11}=yz\times M_{H}+z(1-y)\times M_{H}+y(1-z)\times M_{H}+(1-y)(1-z)\times M_{H}$$

(2) $$\begin{array}{ll} E_{12}= & yz\times [(M_{L}-g_{m}\times ({P_{GM}+P}_{CM})-b\times P_{SM})+z(1-y)\times (M_{L}-g_{m}\times ({P_{GM}+P}_{CM}))\\ & +\;y(1-z)\times (M_{L}-g_{m}\times P_{GM}-b\times P_{SM})+(1-y)(1-z)\times (M_{L}-g_{m}\times P_{GM})] \end{array}$$

(3) $$E_{1}=x\times E_{11}+(1-x)\times E_{12}$$

The replicated dynamic sub-equation of X, as shown in Equation (4), may be obtained from Equations (1)–(3) as follows:

(4) $$F\left(x\right)=\frac{d_{x}}{d_{t}}=x\left(E_{11}-E_{1}\right)=x\left(1-x\right)\left(M_{H}-M_{L}+g_{m}P_{GM}{+zg}_{m}P_{CM}+b{yP}_{SM}\right)$$

(5) $$\frac{dF(x)}{dx}=\left(1-2x\right)\left(M_{H}-M_{L}+g_{m}P_{GM}{+zg}_{m}P_{CM}+b{yP}_{SM}\right)$$

**Figure 2:**
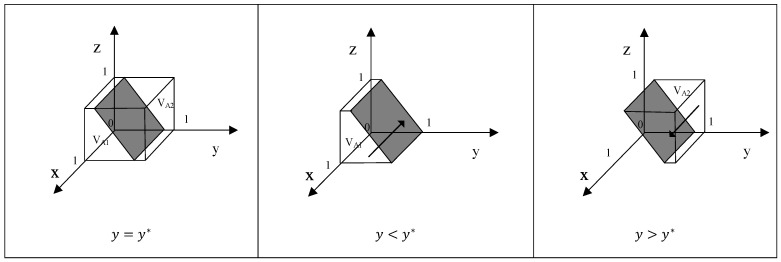
Phase diagram of strategy evolution of behavior of producers.

According to the stability theorem of differential equations, the stabilization strategies of X are determined when the following conditions are simultaneously met: F(x) = 0 and F′(x) = dF(x)/dx < 0. When ${y=y*=(M_{L}-M}_{H}-g_{m}P_{GM}-{zg}_{m}P_{CM})/bP_{SM}$, both F(x) and F′(x) remain constant at 0. This represents the evolutionary stability boundary, where X’s strategic behavior does not change over time. When y>y*, meaning F′(1) < 0 and F′(0) > 0, x = 1 is a stable point. This suggests that when Y’s probability of conducting quality control is higher than y*, the expected economic benefit for X from producing safe food surpasses that of producing substandard food. Conversely, when y<y*, meaning F′(1) > 0 and F′(0) < 0, x = 0 becomes the stable point. This implies that when Y’s probability of conducting quality control is lower than y*, X’s expected profit from producing substandard food is higher, making substandard food production the evolutionarily stable strategy. The evolutionary strategy diagram for this phase is shown in Figure 2, with the arrows indicating X’s progression toward either x = 0 or x = 1.

**Proposition** **1.** *The probability of a producer complying with food safety standards is negatively affected by factors such as the government’s inspection probability, the seller’s ability to detect substandard products, consumers’ ability to make informed consumption choices, and the severity of penalties imposed by both the government and the market when substandard products are detected.*

**Proof.**  Consider the equation ${y*=(M_{L}-M}_{H}-g_{m}P_{GM}-{zg}_{m}P_{CM})/bP_{SM}$. As the values of $g_{m}$, $P_{GM}$, $P_{CM}$, $P_{SM}$, and b,z increase, probability y* decreases, which leads to an expansion in the volume of region $V_{A2}$, thereby increasing the likelihood of producers producing safe food. Conversely, as the value of $M_{L}$ increases, probability y* increases, resulting in a reduction in the volume of region $V_{A2}$, which, in turn, decreases the likelihood of producers producing safe food. □

### 3.2. Analysis of the Evolutionary Strategies of Sellers

Correspondingly, the replicated dynamic sub-equation of Y, as shown in Equation (6), is

(6) $$F\left(y\right)=\frac{d_{y}}{d_{t}}=y\times \left(E_{21}-E_{2}\right)=y\left(1-y\right)\left[S_{H}+\left(x-2\right)C_{M}+\left(1-x\right)bP_{SM}+zm-S_{L}+g_{s}P_{GS}+zg_{s}P_{CS}\right]$$

(7) $$\frac{dF(y)}{dy}=\left(1-2y\right)\left[S_{H}+{(x-2)C}_{M}+\left(1-x\right)bP_{SM}+zm-S_{L}+g_{s}P_{GS}+zg_{s}P_{CS}\right]$$

**Figure 3:**
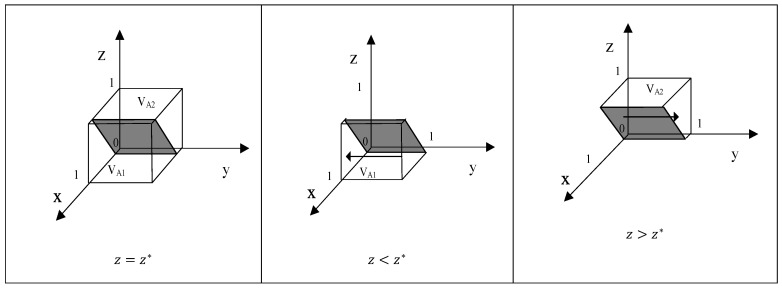
Phase diagram of strategy evolution of behavior of sellers.

According to the stability theorem of differential equations, the stabilization strategies of Y are determined when the following conditions are simultaneously met: F(y) = 0 and F′(y) = dF(y)/dy < 0. When ${z=z*=(S}_{L}-S_{H}-(x-2)C_{M}-(1-x)bP_{SM}-g_{s}P_{GS})/(m+g_{s}P_{CS})$, F(y) is constant 0, and F′(y) is constant 0. This is the evolutionary stability boundary, where Y’s behavioral strategy choice does not change over time. When z>z*, meaning F′(1) < 0 and F′(0) > 0, y = 1 is a stable point. In this case, when Z’s probability of engaging in scientific consumption substitution is higher than z*, the expected economic benefit for Y from conducting quality control surpasses the benefit without quality control. Conversely, when z<z*, meaning F′(1) > 0 and F′(0) < 0, y = 0 becomes the stable point. The evolutionary strategy diagram is shown in Figure 3, with the arrows indicating X’s progression toward either y = 0 or y = 1.

**Proposition** **2.** *Sellers are positively incentivized to engage in quality control by factors such as the government’s probability of sampling inspections, the severity of government penalties and market sanctions for violations, and the price premium consumers are willing to pay for safe food. In contrast, the cost of quality control investments serves as a negative incentive.*

**Proof.**  See the arguments for Proposition 1, above. □

### 3.3. Analysis of the Evolutionary Strategies of Consumers

Correspondingly, the replicated dynamic sub-equation of Z, as shown in Equation (8), is

(8) $$F\left(z\right)=\frac{d_{z}}{d_{t}}=z\times \left(E_{31}-E_{3}\right)\;=z(z-1)(C1+C3-S1+S2-aC1-xC3-yC1+yC2+ayC1-ayC2)$$

(9) $$\frac{dF(z)}{dz}=\left(2z-1\right)\left(C1+C3-S1+S2-aC1-xC3-yC1+yC2+ayC1-ayC2\right)$$

**Figure 4:**
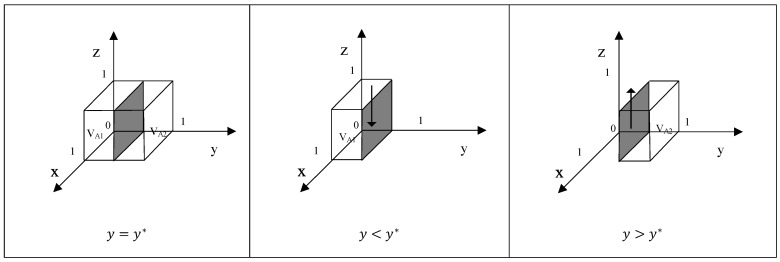
Phase diagram of strategy evolution of behavior of consumers.

According to the stability theorem of differential equations, the stabilization strategies of Z are determined when the following conditions are simultaneously met: F(z)=0 and $F\prime \left(z\right)=\frac{dF(z)}{dz}<0$. When $y=y*=\left[(1-a)C1+(1-x)C3-S1+S2\right]/(1-a)(C1-C2)$, F(z) is constant 0, and F′(z) is constant 0. This is the evolutionary stability boundary, where Z’s behavioral strategy choice does not change over time. When y>y*, meaning F′(1) < 0 and F′(0) > 0, z = 1 is a stable point. In this case, when Y’s probability of conducting quality control is higher than y*, the expected economic benefit for Z from engaging in scientific consumption substitution is higher. Conversely, when y<y*, meaning F′(1) > 0 and F′(0) < 0, z = 0 becomes the stable point, indicating that Z’s expected economic benefit from not making informed consumption substitutions is greater. The evolutionary strategy diagram is shown in Figure 4, with the arrows indicating X’s progression toward either Z = 0 or Z = 1.

**Proposition** **3.** *The matching degree between government-disclosed information and consumer demand positively incentivizes scientific consumption substitution, whereas the information acquisition cost for alternative food quality discourages such substitution.*

**Proof.**  See the arguments for Proposition 1, above. □

### 3.4. Stability Analysis of System Equilibrium Points

As mixed strategy equilibria in asymmetric dynamic games cannot be evolutionarily stable [43], this paper focuses solely on the pure strategy equilibrium points of the evolutionary game system. By setting F(x) = 0, F(y) = 0, and F(z) = 0, eight pure strategy equilibrium points are identified: E1(0, 0, 0), E2(0, 0, 1), E3(0, 1, 0), E4(1, 0, 0), E5(1, 1, 0), E6(1, 0, 1), E7(0, 1, 1), and E8(1, 1, 1). To analyze the stability of these equilibrium points, Ljapunov’s first method (indirect method) is employed by first constructing the Jacobian matrix:

$$J=\left[\begin{array}{ccc} \partial F(x)/\partial x & \partial F(x)/\partial y & \partial F(x)/\partial z\\ \partial F(y)/\partial x & \partial F(y)/\partial y & \partial F(y)/\partial z\\ \partial F(z)/\partial x & \partial F(z)/\partial y & \partial F(z)/\partial z \end{array}\right]=\left[\begin{array}{ccc} a_{11} & a_{12} & a_{13}\\ a_{21} & a_{22} & a_{23}\\ a_{31} & a_{32} & a_{33} \end{array}\right]=\;\left[\begin{array}{ccc} \left(1-2x\right)\left[\begin{array}{c} M_{H}+g_{m}P_{GM}-M_{L}\\ +zg_{m}P_{CM}+b{yP}_{SM} \end{array}\right] & x(1-x)bP_{SM} & x(1-x)g_{m}P_{CM}\\ y(1-y)\left[C_{M}-bP_{SM}\right] & \left(1-2y\right)\left[\begin{array}{c} S_{H}+(x-2)C_{M}\\ +(1-x)bP_{SM}+zm\\ -S_{L}+g_{s}P_{GS}+zg_{s}P_{CS} \end{array}\right] & y(1-y)\left[{m+g}_{s}P_{CS}\right]\\ z(1-z)C3 & z(1-z)(1-a)(C1-C2) & \left(2z-1\right)\left[\begin{array}{c} y(1-a)(C2-C1)+(1-a)C1\\ +(1-x)C3-S1+S2 \end{array}\right] \end{array}\right]$$

On the basis of Lyapunov stability theory [44], when the characteristic values (λ) of the Jacobian matrix satisfy the condition λ < 0, the equilibrium points are in asymptotic stability. Based on the research hypotheses of this study, the eigenvalues, λ, of the Jacobian matrix at each equilibrium point are shown in Table 3.

To facilitate analysis of eigenvalue signs across equilibrium points while maintaining generality, we assume scenarios where $M_{H}-M_{L}+g_{m}(P_{CM}+P_{GM})>0$, $S_{H}-2C_{M}+bP_{SM}-S_{L}+g_{s}P_{GS}+m+g_{s}P_{CS}>0$, and S1−S2+(a−1)C2−C3>0—that is, the net benefits of collaborative governance between consumers and food sellers exceed the total benefits of non-cooperative outcomes. Given the model’s parametric complexity, we examine three representative cases to derive evolutionarily stable strategies (ESSs).

Case 1: When $S_{H}-C_{M}-S_{L}+g_{s}P_{GS}+m+g_{s}P_{CS}>0$ and S1−S2−(1−a)C1>0, the seller’s payoff from implementing quality control exceeds that from non-compliance when detected by government sampling, and the cost of acquiring information is lower than the resulting gain in consumer surplus. As shown in Table 4, under these conditions, both equilibrium points A1(0, 0, 0) and A8(1, 1, 1) have Jacobian matrices with non-positive eigenvalues.
Case 2: When $S_{H}-C_{M}-S_{L}+g_{s}P_{GS}+m+g_{s}P_{CS}>0$ and S1−S2−(1−a)C1<0, the seller’s total payoff from quality control exceeds that from non-compliance under government sampling, while the consumer’s information cost exceeds the resulting gain in consumer surplus. As shown in Table 4, the Jacobian matrices at equilibrium points E1(0, 0, 0), E4(1, 0, 0), and E8(1, 1, 1) all have non-positive eigenvalues.
Case 3: When $S_{H}-C_{M}-S_{L}+g_{s}P_{GS}+m+g_{s}P_{CS}<0$ and S1−S2−(1−a)C1>0, the seller’s total payoff from quality control is lower than that from not implementing it when detected by government sampling, while the consumer’s cost of acquiring substitute information is lower than the corresponding gain in consumer surplus from informed decision-making. As shown in Table 4, the Jacobian matrices at equilibrium points A1(0, 0, 0) and A6(1, 0, 1) have non-positive eigenvalues.

### 3.5. Measuring the Alignment Between Government Information Disclosure and Consumer Information Needs

Based on existing studies, this paper measures information alignment from four dimensions: timeliness, content, channels, and format of government disclosure. In the questionnaire, respondents rated the degree of mismatch in these four dimensions (using items from Table 5) on a five-point Likert scale, where higher scores indicate greater misalignment. This study collected data through the SoJump and Credamo online survey platforms.

In the absence of clear theoretical or empirical justification for differential weighting across dimensions, equal weights are assigned to the four dimensions. The composite mismatch score, a′∈ [1, 5], is computed as the mean of the dimensional mismatch scores. To improve interpretability, a′ is linearly transformed into the interval [1, 10]:

(10) $$a_{x}=\frac{10-1}{5-1}\left(a\prime -1\right)+1$$

where $a_{x}$ represents the misalignment between government food safety disclosure and consumer needs for respondents aware of such disclosures. For consumers unaware of government disclosures but who actively seek food safety information in daily life, misalignment arises primarily from ineffective dissemination; their score is, therefore, set to the maximum value, $a_{x}$ = 10. The overall misalignment score, a″, is then calculated as the weighted average of $a_{x}$ and 10, using the respective sample proportions. The alignment index is defined as

(11) a=(1−a″)/10

Higher a values indicate greater alignment between government-disclosed information and consumer needs.

## 4. Numerical Simulation Analysis

To validate the theoretically derived stability points of the social co-governance system, numerical simulations were conducted using Matlab R2021a to examine how key factors influence stakeholder strategies and guide the system toward desirable equilibria. Based on the research assumptions and Table 3, three parameter sets and their justifications are presented in Table 6. Key parameter values were determined not only from the literature but also through consumer surveys, expert interviews, and field investigations. To assess the alignment between government food inspection disclosures and consumer information needs, 882 valid online survey responses were collected. Detailed survey results are provided in the Supplementary Materials. For parameters lacking direct empirical references, values were finalized through iterative simulations and practical validation.

### 4.1. Numerical Simulation Under Varying Initial Willingness

**Figure 5:**
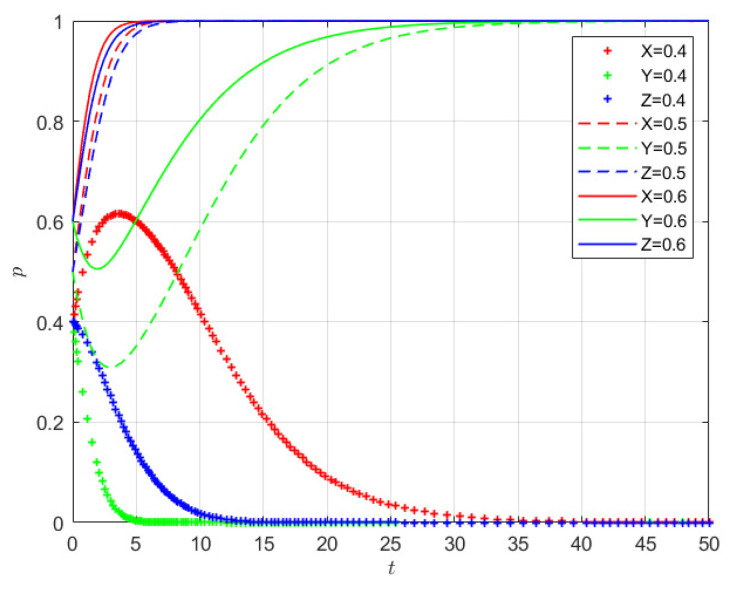
Evolution of the system under varying initial willingness levels of all three stakeholders.

Based on the parameter values defined for each scenario, while holding other variables constant, this section explores how variations in the initial willingness of producers, sellers, and consumers affect the level of coordinated quality control within the supply chain. Using the parameter values from Scenario 2 as an example, the corresponding evolutionary trajectories are shown in Figure 5. As illustrated, when the initial willingness of X, Y, and Z is at or above the threshold of 0.5, all three variables rapidly converge to 1. The system ultimately reaches the equilibrium point E8(1, 1, 1), indicating full participation by producers, sellers, and consumers. Moreover, the higher the initial willingness, the faster the convergence. Notably, producers and consumers reach the equilibrium state more quickly than sellers. Conversely, when the initial willingness of X, Y, and Z is below 0.5, all three variables rapidly converge to 0, suggesting complete withdrawal from cooperation. In this case, sellers exhibit a faster rate of convergence compared to producers and consumers.

### 4.2. Impact of Key Parameter Values on Participants’ Behavior Strategies

#### 4.2.1. Impact of Information Alignment on Strategy Choices of Participants

**Figure 6:**
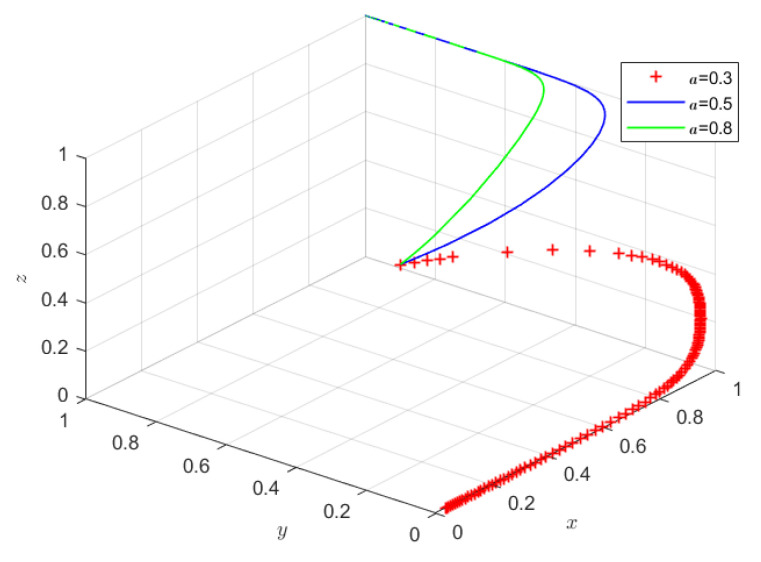
The impact of information matching (a) on system evolution.

Holding all other parameters constant, Scenario 2 sets the initial willingness of producers, sellers, and consumers to (0.5, 0.5, 0.5). Based on consumer survey results, three levels of alignment between government food safety disclosures and consumer needs were simulated: 0.3, 0.5, and 0.8. According to Figure 6, when government-disclosed food safety inspection information poorly aligns with consumers’ information needs, the cost of acquiring risk information across substitute food products is relatively high. After weighing information acquisition costs against expected consumption utility, rational consumers are unlikely to attend to or use such information in their decision-making. As a result, consumption choices may be scientifically suboptimal, making it difficult to generate coordinated scientific consumption substitution among consumers. In the absence of market incentives, producers’ strategies converge toward producing risky food, while sellers evolve toward non-participation in quality control, leading the system to the equilibrium E1(0,0,0), where social co-governance fails to emerge.

As information alignment improves, the cost of acquiring risk information across substitutes decreases, encouraging consumers to engage in informed substitution. In response, sellers evolve toward participating in quality control, and producers shift toward producing safe food, driving the system toward the equilibrium E8(1,1,1) and enhancing the level of social co-governance. Further improvements in information alignment accelerate the convergence of consumers, sellers, and producers’ strategies toward co-governance. These findings are consistent with Chen et al. (2025) [46], who show that higher information-matching efficiency strengthens the effect of quality signals in mitigating adverse selection in agri-food markets, and they further extend the conclusions of Luo et al. (2019) [33] and Kariuki & Hoffmann (2021) [25].

#### 4.2.2. Sellers’ Effective Inspection Rates on the Behavioral Strategy Choices of Participating Entities

**Figure 7:**
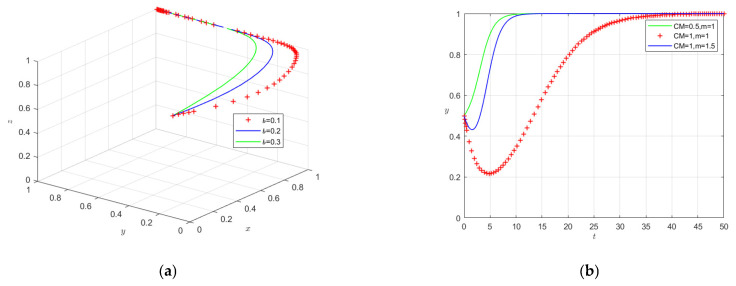
(**a**) Effect of effective inspection rate (b) on system evolution; (**b**) influence of quality control costs ($C_{M}$) and market incentives (*m*) on seller behavior decisions.

Assuming constant values for all other parameters, we set sellers’ effective inspection rates at 0.1, 0.2, and 0.3. Figure 7a presents the simulation results of strategy selection among the stakeholders. The results indicate that enhancing sellers’ effective inspection capacity accelerates the evolutionary convergence toward social co-governance, consistent with the findings of Wu et al. (2024) [4]. These findings highlight the importance of technical support and policy incentives to help sellers improve inspection capabilities. Strategic decisions in enterprises are also influenced by cost–benefit dynamics. As shown in Figure 7b, increasing market incentives by 0.5 units (e.g., from 1.0 to 1.5) and reducing quality control costs by 0.5 units (e.g., from 1.0 to 0.5) both facilitate the adoption of quality control. However, cost reductions lead to faster strategy shifts than equivalent incentive increases, suggesting that lowering quality control costs is a more effective short-term measure.

#### 4.2.3. Impact of Government Sampling and Information Disclosure Policy Combinations on Stakeholders’ Behavioral Strategy Choices

**Figure 8:**
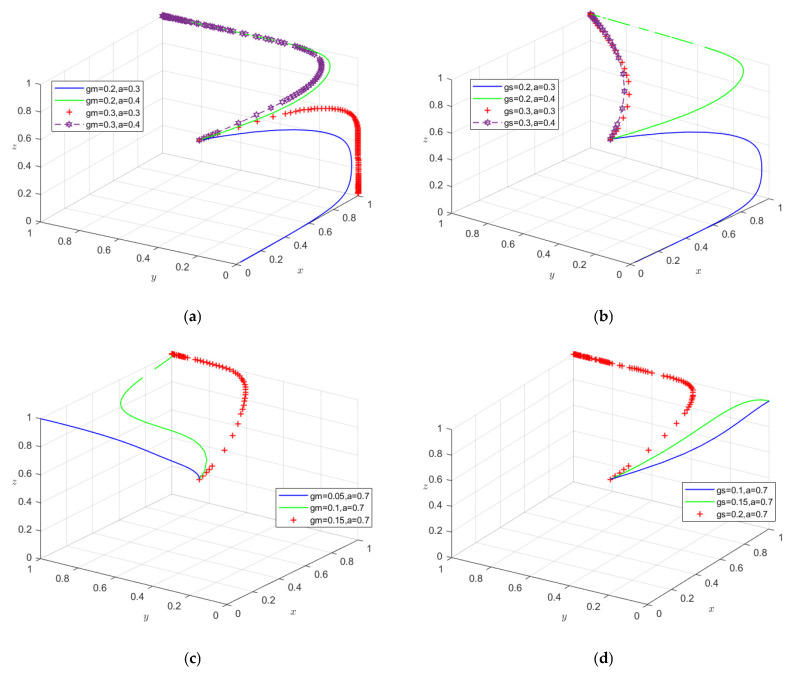
(**a**) Impact of government regulation of producers ($g_{m}$) combined with low-level information alignment (a) on system evolution; (**b**) impact of government regulation of sellers ($g_{S}$) combined with low-level information alignment (a) on system evolution; (**c**) impact of government regulation of producers ($g_{m}$) combined with high-level information alignment (a) on system evolution; (**d**) impact of government regulation of sellers ($g_{S}$) combined with high-level information alignment (a) on system evolution.

This study further examines how government sampling intensity and the alignment between information disclosure and consumer demand influence system evolution. Holding other parameters constant, stakeholders’ initial willingness in Scenario 2 is set at (0.5, 0.5, 0.5). When the alignment between government food inspection disclosures and consumer needs is low, improving alignment yields greater marginal gains in promoting social co-governance. Specifically, a 0.1-unit increase in information alignment enhances social co-governance more than an equivalent increase in producer inspection intensity (Figure 8a). However, under the same conditions, a 0.1-unit increase in seller inspection intensity produces a greater improvement than the same increase in information alignment (Figure 8b). This indicates that when disclosure effectiveness is limited, strengthening seller inspections is more effective than increasing producer inspections for rapidly advancing social co-governance. Importantly, raising inspection intensity incurs higher regulatory costs, whereas improving information alignment is more cost-effective and sustainable.

Simulation results (Figure 8c,d) show that even with high alignment between government disclosures and consumer needs, insufficient inspection of producers and sellers weakens policy synergy and hinders coordinated supply chain quality control. This suggests that the empowering effect of inspection disclosures on social co-governance relies heavily on adequate enforcement. Under these conditions, increasing inspection intensity on producers has a stronger impact on social co-governance than increasing seller inspections, highlighting the institutional advantage of source-oriented governance. Integrating results from Figure 8a–d shows that combining higher inspection intensity with improved information alignment accelerates social co-governance and achieves stronger food safety governance than single-policy approaches, consistent with Zhou et al. (2022) [2]. Accordingly, governments should maintain sufficient inspection intensity on producers and sellers while ensuring high alignment between disclosed information and consumer needs. Regulatory priorities can then be dynamically adjusted across stages to optimize resource allocation and efficiently enhance overall social co-governance.

## 5. Main Conclusions and Recommendations

### 5.1. Conclusions and Discussion

To address challenges in food safety social co-governance—such as limited public participation and weak effects of government information disclosure—this study develops a three-stage evolutionary game model involving food producers, sellers, and consumers. Drawing on a government–market collaborative governance framework and the perspectives of information asymmetry and alignment, the model systematically examines the interlinked incentives among stakeholders. It highlights how food safety information disclosure activates market-driven mechanisms and strengthens supply chain quality control. Based on this analysis, the following conclusions are drawn:

First, insufficient alignment between government-disclosed food safety inspection information and consumer needs limits public engagement and the effectiveness of social co-governance. Model results indicate that only when disclosure aligns with consumer preferences in content, channels, and presentation—thereby lowering information acquisition costs—can acceptance and use of inspection results be enhanced. This promotes informed consumption substitution and market discipline through “voting with their feet,” encouraging producers and sellers to implement quality control and facilitating food safety co-governance.

Second, reducing quality control costs for sellers is more effective in the short term than increasing market incentives in promoting the adoption of quality control measures. The study shows that while both a 0.5-unit increase in market incentives and a 0.5-unit reduction in quality control costs encourage sellers to adopt quality control strategies, the cost reduction has a more immediate impact.

Third, a combined policy of enhancing inspection intensity and improving information alignment achieves social co-governance more rapidly than either policy alone. Further analysis shows that when information alignment is low, improving alignment offers higher cost-effectiveness and sustainability, and strengthening the inspection of sellers is more effective than targeting producers in quickly advancing co-governance. Conversely, when information alignment is high, intensifying inspections of producers more effectively accelerates social co-governance.

### 5.2. Policy Recommendations

1. Optimize food safety inspection disclosure and enhance alignment with consumer needs. The government should implement a demand-driven disclosure mechanism that collects consumer concerns about food categories, sellers, and purchase channels through surveys and interactive platforms (e.g., “Click and Inspect”). Disclosure content should be dynamically adjusted to meet diverse public needs. Leveraging new media, a coordinated “government–media” communication system can convey risk differences among substitute products to a broader audience. To reduce information processing costs, disclosure should prioritize visualization and interactivity, enhancing readability and comprehension. Furthermore, big data analytics can extract insights from inspection records across food types, sellers, channels, and regions, assess relative risks among substitutes, support consumers in informed substitution choices, and help the government optimize regulatory resource allocation.
2. Strengthen sellers’ quality control incentives and lower implementation barriers. To motivate sellers effectively in the short term, the government should complement improved disclosure with targeted support, such as subsidies for testing equipment and reagents. Enhancing sellers’ training in quality management can improve self-inspection capabilities and standardization. Public education on food safety risks can raise consumer awareness, reducing the cost of accessing risk information among substitute products and promoting rational substitution choices, thereby reinforcing the market-driven mechanism. Building on this, greater use should be made of third-party platforms and market mechanisms in quality and safety governance. Strengthening e-commerce platforms’ roles in food safety review, disclosure, and traceability, together with standardizing the use of safety labels, quality certifications, and traceability marks by offline sellers, can reinforce market incentives for quality control through institutionalized quality signals.
3. Enhance the synergy between inspection and information disclosure to optimize regulatory resource allocation. Building on high disclosure alignment, inspection intensity should be increased to create complementary effects, achieving a “1 + 1 > 2” impact on food safety governance. When disclosure alignment is low, priority should be given to inspecting sellers to quickly improve overall system coordination. Once disclosure alignment improves, regulatory focus should shift to the production stage to maximize governance efficiency and sustainability.
4. Strengthen consumer education to promote informed choices. Consumers should receive education on food safety and quality to enhance their risk awareness. In practice, the most visually appealing foods are not necessarily the highest quality. Through targeted outreach and training, consumers can be guided to make informed decisions based on inspection results and disclosed information, thereby fostering a positive interaction among government supervision, producer responsibility, and consumer behavior.

Although this study provides an in-depth analysis of the role of government food safety information alignment in enabling food safety co-governance—and its findings are applicable to food safety regulatory contexts in other countries—it nonetheless has the following limitations. First, constrained by the evolutionary game approach, the simulations—though informed by consumer surveys, expert interviews, and the existing literature—still lack extensive practical validation. Future research could integrate surveys and choice experiments to provide more targeted empirical support. Second, this study does not identify which inspection content, presentation formats, or dissemination channels most effectively promote rational consumer substitution, calling for further investigation. Third, the analysis is limited to production and sales stages, excluding material supply and logistics. Future studies could extend the framework to encompass suppliers, logistics firms, and other actors across the entire food supply chain.

## Figures and Tables

**Table 1:** Parameters and their meanings.

Symbols	Meanings and Explanation
*x*	Probability that X chooses to produce safe food, 0 ≤ *x* ≤ 1
*y*	Probability that Y chooses to sell safe food, 0 ≤ *y* ≤ 1
*z*	Probability that Z engages in scientific consumption substitution, 0 ≤ *z* ≤ 1
$M_{H}$	Profit for X from producing safe food
$M_{L}$	Profit for X from producing substandard food
$g_{m}$	Probability of government inspection of X
$P_{GM}$	Government penalty on X for violations
$P_{CM}$	Market penalty by Z on X for violations
$S_{H}$	Profit for Y from conducting quality control
$S_{L}$	Profit for Y from not conducting quality control
$C_{M}$	Cost incurred by Y for inspecting X’s food
b	Probability that substandard food is detected when Y inspects, 0 ≤ b ≤ 1
$P_{SM}$	Penalty imposed by Y on X for substandard food
*m*	Market reward for Y from Z for selling safe food
$g_{S}$	Probability of government inspection of Y
$P_{GS}$	Penalty imposed by the government on Y for violations
$P_{CS}$	Market penalty imposed by Z on Y for violations
*S*1	Consumer surplus when Z engages in scientific consumption substitution
*S*2	Consumer surplus when Z does not engage in scientific consumption substitution
*C*1	Information acquisition cost borne by Z when government disclosure is perfectly mismatched
*C*2	Information acquisition cost borne by Z under perfect disclosure mismatch from both government and sellers
*C*3	Switching cost incurred by Z when engaging in consumption substitution
a	Degree of alignment between government-disclosed information and consumer needs

**Table 2:** The payoff matrix of the three game participants.

Producers	Consumers	Sellers	
		Conducting Quality Control	No Quality Control
Producing safe food	Scientific consumption substitution	$M_{H}$	$M_{H}$
		$S_{H}-C_{M}+m$	$S_{L}-g_{S}\times (P_{GS}+P_{CS})$
		S1−(1−a)×C2	S1−(1−a)×C1
	Non-participation	$M_{H}$	$M_{H}$
		$S_{H}-C_{M}$	$S_{L}-g_{S}\times P_{GS}$
		S2	S2
Producing substandard food	Scientific consumption substitution	$M_{L}-g_{m}\times ({P_{GM}+P}_{CM})-b\times P_{SM}$	$M_{L}-g_{m}\times (P_{GM}+P_{CM})$
		$S_{H}-{2C}_{M}+b\times P_{SM}+m$	$S_{L}-g_{S}\times (P_{GS}+P_{CS})$
		S1−(1−a)×C2−C3	S1−(1−a)×C1−C3
	Non-participation	$M_{L}-g_{m}\times P_{GM}-b\times P_{SM}$	$M_{L}-g_{m}\times P_{GM}$
		$S_{H}-{2C}_{M}+b\times P_{SM}$	$S_{L}-g_{S}\times P_{GS}$
		S2	S2

**Table 3:** Characteristic values of the Jacobian matrix.

Equilibrium Point	λ1	λ2	λ3
E1(0, 0, 0)	$M_{H}-M_{L}+g_{m}P_{GM}$	$S_{H}-2C_{M}+bP_{SM}-S_{L}+g_{s}P_{GS}$	S1−S2+(a−1)C1−C3
E2(0, 0, 1)	$M_{H}-M_{L}+g_{m}{(P}_{CM}+P_{GM})$	$S_{H}-2C_{M}+bP_{SM}-S_{L}+g_{s}P_{GS}+m+g_{s}P_{CS}$	−S1+S2+(1−a)C1+C3
E3(0, 1, 0)	$M_{H}-M_{L}+g_{m}P_{GM}+bP_{SM}$	$-S_{H}+2C_{M}-bP_{SM}+S_{L}-g_{s}P_{GS}$	S1−S2+(a−1)C2−C3
E4(1, 0, 0)	${-M}_{H}+M_{L}-g_{m}P_{GM}$	$S_{H}-C_{M}-S_{L}+g_{s}P_{GS}$	S1−S2+(a−1)C1
E5(1, 1, 0)	${M_{L}-M}_{H}-g_{m}P_{GM}-bP_{SM}$	${C_{M}-S}_{H}+S_{L}-g_{s}P_{GS}$	S1−S2+(a−1)C2
E6(1, 0, 1)	${M_{L}-M}_{H}-g_{m}{(P}_{CM}+P_{GM})$	$S_{H}-C_{M}-S_{L}+g_{s}P_{GS}+m+g_{s}P_{CS}$	−S1+S2+(1−a)C1
E7(0, 1, 1)	$M_{H}-M_{L}+g_{m}{(P}_{CM}+P_{GM})+bP_{SM}$	$2C_{M}-S_{H}+S_{L}-bP_{SM}-m-g_{s}P_{GS}-g_{s}P_{CS}$	−S1+S2+(1−a)C2+C3
E8(1, 1, 1)	${M_{L}-M}_{H}-g_{m}{(P}_{CM}+P_{GM})-bP_{SM}$	${C_{M}-S}_{H}+S_{L}-g_{s}P_{CS}-m-g_{s}P_{GS}$	−S1+S2+(1−a)C2

**Table 4:** Equilibrium point stability analysis of the system.

Equilibrium Point	Case 1				Case 2				Case 3			
	λ1	λ2	λ3	Stability Conditions	λ1	λ2	λ3	Stability Conditions	λ1	λ2	λ3	Stability Conditions
E1(0, 0, 0)	+, −	+, −	+, −	ESS or Unstable	+, −	+, −	−	ESS or Unstable	+, −	+, −	+, −	ESS or Unstable
E2(0, 0, 1)	+	+	+, −	Unstable	+	+	+	Saddlepoint	+	+	+, −	Unstable
E3(0, 1, 0)	+, −	+, −	+	Unstable	+, −	+, −	+	Unstable	+, −	+, −	+	Unstable
E4(1, 0, 0)	+, −	+, −	+	Saddlepoint or Unstable	+, −	+, −	−	ESS or Unstable	+, −	+, −	+	Saddlepoint or Unstable
E5(1, 1, 0)	+, −	+, −	+	Saddlepoint or Unstable	+, −	+, −	+	Saddlepoint or Unstable	+, −	+, −	+	Saddlepoint or Unstable
E6(1, 0, 1)	−	+	−	Unstable	−	+	+	Unstable	−	−	−	ESS
E7(0, 1, 1)	+	−	−	Unstable	+	−	−	Unstable	+	−	−	Unstable
E8(1, 1, 1)	−	−	−	ESS	−	−	−	ESS	−	+	−	Unstable

**Table 5:** Measurement scale for information matching degree.

Dimension	Specific Questions
Timeliness	Compared to my needs, I think the current frequency of government disclosure of food sampling information is even slower.
Content	It’s a struggle to take the food safety information released by the government and process it into the information I want.
Channels	Official channels such as government websites/public numbers are not my main channels for obtaining food safety information.
Format	The current presentation of sampling information does not make it easy for me to find key risk information.

**Table 6:** Assigned parameter values for the three cases.

Parameters	Case 1	Case 2	Case 3	Basis for Parameter Assignment
$M_{H}$	6	6	6	Wan et al. (2018) [40]
$M_{L}$	8	8	8	
$S_{H}$	4	4	4	
$S_{L}$	6	6	6	
$C_{M}$	1	1	1	
b	0.2	0.2	0.4	
$P_{GM}$	9	9	9	
$P_{GS}$	7	7	7	
$P_{SM}$	6	6	6	
$g_{m}$	0.2	0.2	0.2	
$g_{S}$	0.2	0.2	0.1	
$P_{CM}$	4	4	4	Gao et al. (2023) [7]
$P_{CS}$	4	4	4	
*m*	1	1	1	Expert interview
*S*1	6	6	6	Wu et al. (2024) [4], Gao et al. (2023) [7]
*S*2	4	4	4	
*C*1	2.5	3	2	Expert interview, parameter refinement and testing
*C*2	1	1.5	1	Expert interview, parameter refinement and testing
*C*3	0.7	0.7	0.7	Jin et al. (2015) [45]
a	0.3	0.7	0.3	Consumer survey data

## Data Availability

The original contributions presented in the study are included in the article and Supplementary Materials. Further inquiries can be directed to the corresponding author.

## Supplementary Materials

The following supporting information can be downloaded at: https://www.mdpi.com/article/10.3390/foods15050876/s1.

## References

[1] World Health Organization (2020). Meeting Report: The Second Global Meeting of the FAO/WHO International Food Safety Authorities Network (INFOSAN).

[2] Zhou J., Jin Y., Liang Q., Kydd J. (2022). Effects of regulatory policy mixes on traceability adoption in wholesale markets: Food safety inspection and information disclosure. Food Policy.

[3] Barnes J., Whiley H., Ross K., Smith J. (2022). Defining food safety inspection. Int. J. Environ. Res. Public Health.

[4] Wu L., Ling Z., Chen X. (2024). Selection of behavior strategy for quality investment in food supply chain under social Co-governance famework. J. Macro-Qual. Res..

[5] Xie K., Lai J., Xiao J., Wu J. (2016). Food safety, regulatory boundaries, and institutional arrangements. Econ. Res. J..

[6] Martinez M.G., Fearne A., Caswell J.A., Henson S. (2007). Co-regulation as a possible model for food safety governance: Opportunities for public–private partnerships. Food Policy.

[7] Gao H., Dai X., Wu L., Zhang J., Hu W. (2023). Food safety risk behavior and social Co-governance in the food supply chain. Food Control.

[8] Unnevehr L.J. (2007). Food safety as a global public good. Agric. Econ..

[9] Zhou J., Hu J. (2009). Analysis of quality and safety management practices of vegetable processing enterprises and their influencing factors—A case study of Zhejiang. Chin. Rural Econ..

[10] Xu J., Cai J., Yao G., Dai P. (2022). Strategy Optimization of Quality Improvement and Price Subsidy of Agri-Foods Supply Chain. Foods.

[11] Wu Y. (2012). Information foundation, reputation mechanism, and law enforcement optimization -A new perspective on food safety governance. Soc. Sci. China.

[12] Gong Q., Zhang Y., Yu J. (2013). Incentives, information and food safety regulation. Econ. Res. J..

[13] Henson S., Caswell J. (1999). Food safety regulation: An overview of contemporary issues. Food Policy.

[14] Su Y., Yu H., Wang M., Li X., Li Y. (2022). Why did China’s cost-reduction-oriented policies in food safety governance fail? The collective action dilemma perspective. Can. J. Agric. Econ..

[15] Wang C., Gu H. (2012). Are Chinese consumers forgetful?—An economic analysis of Chinese consumer tolerance towards corporate food safety issues. Res. Econ. Manag..

[16] Jin H.J., Han D.H. (2014). Interaction between message framing and consumers’ prior subjective knowledge regarding food safety issues. Food Policy.

[17] Thaler R.H., Sunstein C.R. (2008). Nudge: Improving Decisions About Health, Wealth, and Happiness.

[18] Meng X., Qian K., Li T. (2021). Study on the safe consumption strategy of food and agricultural products in China under the perspective of consumption substitution. Dongyue Trib..

[19] Self D., Rothstein H. (2021). Institutional constraints on ‘nudge-style’ risk rating systems: Explaining why food hygiene barometers were rolled-out in the UK but abandoned in Germany. J. Risk Res..

[20] Etzioni A. (2010). Is transparency the best disinfectant?. J. Political Philos..

[21] Haufler V. (2010). Disclosure as governance: The extractive industries transparency initiative and resource management in the developing world. Glob. Environ. Politics.

[22] Ollinger M., Bovay J. (2020). Producer response to public disclosure of food-safety information. Am. J. Agric. Econ..

[23] Arnade C., Calvin L., Kuchler F. (2009). Consumer response to a food safety shock: The 2006 food-borne illness outbreak of *E. coli* O157: H7 linked to spinach. Rev. Agric. Econ..

[24] Toledo C., Villas-Boas S.B. (2019). Safe or not? Consumer responses to recalls with traceability. Appl. Econ. Perspect. Policy.

[25] Kariuki S.W., Hoffmann V. (2021). Can information drive demand for safer food? Impact of brand-specific recommendations and test results on product choice. Agric. Econ..

[26] Bovay J. (2022). Food safety, reputation, and regulation. Appl. Econ. Perspect. Policy.

[27] Lin W., Ma B., Liang J., Jin S. (2024). Price response to government disclosure of food safety information in developing markets. Food Policy.

[28] Jin G.Z., Leslie P. (2003). The effect of information on product quality: Evidence from restaurant hygiene grade cards. Q. J. Econ..

[29] Jin G.Z., Leslie P. (2019). New evidence on information disclosure through restaurant hygiene grading: Reply. Am. Econ. J. Econ. Policy.

[30] Choi J., Scharff R.L. (2017). Effect of a publicly accessible disclosure system on food safety inspection scores in retail and food service establishments. J. Food Prot..

[31] Dillaway R., Messer K.D., Bernard J.C., Kaiser H.M. (2011). Do consumer responses to media food safety information last?. Appl. Econ. Perspect. Policy.

[32] Hoffmann V., Moser C.M., Herrman T.J. (2021). Demand for aflatoxin-safe maize in Kenya: Dynamic response to price and advertising. Am. J. Agric. Econ..

[33] Luo J., Chen T., Pan J. (2019). Evolutionary dynamics of health food safety regulatory information disclosure from the perspective of consumer participation. Food Sci. Nutr..

[34] Wang L., Demeritt D., Rothstein H. (2023). “Carrying the black pot”: Food safety and risk in China’s reactive regulatory state. Regul. Gov..

[35] Zhou X., Yang Y. (2018). Research on the supply of food quality and safety information: Government vs. third-party certification agencies. Theory Pract. Price.

[36] Yang X., Wu X. (2022). Research on the risk reverse evaluation model of food safety information disclosure. Zhejiang Acad. J..

[37] Si X., Huo C., Zhao D. (2024). Exploring information communication gaps between government information supply and public information demand during public crisis. Inf. Stud. Theory Appl..

[38] Viscusi W.K., Magat W.A., Huber J. (1986). Informational regulation of consumer health risks: An empirical evaluation of hazard warnings. RAND J. Econ..

[39] Chen Q., Min C., Zhang W., Wang G., Ma X., Evans R. (2020). Unpacking the black box: How to promote citizen engagement through government social media during the COVID-19 crisis. Comput. Hum. Behav..

[40] Wan C., Qin Z., Wu J. (2018). Research on food safety risk control from the perspective of supply chain. China Soft Sci..

[41] Ortega D.L., Wang H.H., Wu L., Olynk N.J. (2011). Modeling heterogeneity in consumer preferences for select food safety attributes in China. Food Policy.

[42] Liu R., Gao Z., Nayga R.M., Snell H.A., Ma H. (2019). Consumers’ valuation for food traceability in China: Does trust matter?. Food Policy.

[43] Selten R. (1980). A note on evolutionarily stable strategies in asymmetric animal conflicts. J. Theor. Biol..

[44] Parks P.C. (1992). AM Lyapunov’s stability theory—100 years on. IMA J. Math. Control. Inf..

[45] Jin M., Zhao M., Yang B., Zhang Y. (2015). An analysis of consumer substitution intentions under the influence of food safety incidents: A case study of the KFC food safety incident. China Rural. Econ..

[46] Chen J., Lu F., Wang X. (2025). Research on the impact and mechanism of quality signal on adverse selection in edible. Agric. Prod. Mark..

