# Supplementary material for: Quality and Safety Risk Control in the Food Supply Chain: An Information Disclosure Approach to Supply–Demand Alignment
Source: Foods. 2026 Mar 4;15(5):876. doi: 10.3390/foods15050876 (PMC12985150; doi:10.3390/foods15050876)
Supplement: Supplementary file 1 [file foods-15-00876-s001.zip › foods-4057770-supplementary.pdf]

Supplementary Materials

This study collected data through the SoJump and Credamo online survey platforms, yielding a total of 920 responses. After excluding questionnaires with unusually short completion times or incorrect answers to attention-check questions, 882 valid responses remained. Among these, 45% of respondents were male and 55% female; 42% were from rural areas and 58% from urban areas.

The survey first asked: “How often do you view government-released food safety inspection information?” The response options were:“Never (I don't know the government has disclosed the sampling information), hardly ever (I know the government has announced the sampling information but I don't want to view it), about once every six months, about once a month, and about once every one to two weeks”. For those who did not know that the government disclosed information on food sampling, the question was further pressed, “Do you usually pay attention to food safety information actively?”. If the answer is “yes”, the mismatch between the food safety information disclosed by the government and the information demanded by consumers is 1. There are 64 samples in this section.

Respondents rated the perceived mismatch between government food safety information and their needs across four dimensions: timeliness, content, channels, and format. The average mismatch scores were 2.16, 3.89, 3.88, and 3.87, respectively. Assigning equal weights to each dimension, the overall average mismatch score was 3.45 on a 1–5 scale. This was then linearly mapped to a 1–10 scale, resulting in a score of 6.51. Accordingly, the information mismatch for this group was approximated as 0.65.

Finally, a weighted average was calculated based on 64 responses with a mismatch score of 1 and 818 responses with a mismatch score of 0.65, resulting in an overall mismatch score of 0.67, or a matching score of 0.32. In the evolutionary game analysis, this matching degree was approximated as 0.3.
